# Intraoperative model-guided anchor placement with CT verification for non-percutaneous localization of small pulmonary nodules: a retrospective feasibility study

**DOI:** 10.3389/fonc.2026.1941071

**Published:** 2026-10-19

**Authors:** Xining Zhang, Jingwei Liu, Shijie Zhang, Jian Li

**Affiliations:** Peking University First Hospital, Beijing, China

**Keywords:** hybrid operating room, nodule localization, non-percutaneous, pulmonary nodules, VATS

## Abstract

**Background:**

Accurate localization of small pulmonary nodules, particularly ground-glass-dominant and deep lesions, remains challenging in thoracoscopic surgery. We evaluated a model-guided, non-percutaneous approach to intraoperative localization.

**Method:**

We retrospectively reviewed consecutive patients who underwent intraoperative model-guided anchor-based non-percutaneous localization or conventional preoperative CT-guided puncture localization and resection for small pulmonary nodules at our center between January 1 and December 31, 2024. Localization-specific and perioperative outcomes were compared between groups.

**Results:**

Twenty-four patients underwent intraoperative localization, and 91 underwent preoperative puncture localization. Median localization time with the intraoperative approach was 35.7 minutes (IQR 28.8-47.3 minutes). The observed localization-category distributions were similar between groups (precise: 50% vs. 49.49%; helpful: 43.33% vs. 42.42%; missed: 6.67% vs. 8.08%; p = 1.000). In this indication-selected cohort, intraparenchymal hemorrhage (0% vs. 16.16%, p = 0.022) and pneumothorax (0% vs. 31.87%, p < 0.001) were observed less frequently in the intraoperative group. All patients in the intraoperative-localization group subsequently underwent CONSERVO electroresection, whereas the conventional group underwent stapler-based resection. The combined intraoperative localization/CONSERVO strategy was associated with a shorter incision length but a longer non-localization operative time, greater blood loss, a longer chest-tube duration, and greater early drainage. Because localization and resection techniques differed concurrently, their independent contributions could not be determined.

**Conclusion:**

Intraoperative model-guided anchor placement with CT verification was technically feasible in this small retrospective cohort. The approach avoided transthoracic puncture and may warrant further evaluation for selected patients with multiple nodules or lesions unsuitable for preoperative puncture or direct tactile localization.

## Background

With the widespread adoption of low-dose computed tomography (LDCT) for lung cancer screening, the detection rate of pulmonary nodules has increased substantially (1). Current guidelines recommend resection for nodules with a high likelihood of malignancy in the absence of surgical contraindications (2–4). While subpleural or solid-subsolid nodules are often readily localized and resected, ground-glass-dominant or deeply located nodules, particularly those adjacent to the hilum, mediastinum, or diaphragm, remain challenging to localize precisely during video-assisted thoracoscopic surgery (VATS). Reliable and accessible localization methods are therefore essential to optimize oncologic outcomes, minimize unnecessary parenchymal loss, and reduce the need for conversion to thoracotomy.

Current localization options include CT-guided hookwire, microcoil, liquid-marker or ICG injection, bronchoscopic marking, intraoperative ultrasonography, and image-guided techniques performed in a hybrid operating room. Each method has specific advantages and limitations related to transthoracic access, marker stability, lesion location, equipment availability, radiation exposure, and institutional workflow. Although several retrospective cohorts suggest advantages of liquid markers over hookwire for selected outcomes, no single method has been established as universally preferable across clinical settings. For example, preoperative CT-guided hook-wire placement or medical glue injection involves inserting a tangible object into the parenchyma near the nodule, thus facilitating palpation. However, this widely used method is not without limits, as its success rate depends on multiple factors (5) and carries risks of mislocalization, anchor migration, pneumothorax, and bleeding. In rare cases, fatal air embolism can occur (6, 7). Furthermore, transthoracic access may also be difficult or infeasible for lesions near the mediastinum, diaphragm, or interlobar fissures. Intraoperative ultrasonography provides a puncture-free alternative, but localization of pure ground-glass nodules can remain challenging (8).

Hybrid operating-room imaging enables localization and resection to be performed during the same anesthetic episode. However, most reported intraoperative CT-guided methods continue to use percutaneous needle placement (9, 10), whereas localization based solely on preoperative surface projection may be affected by positional and inflation-related deformation of the lung (11–13). Methods capable of combining preoperative spatial planning with intraoperative evaluation while avoiding intentional transthoracic traversal of the lung parenchyma therefore warrant investigation. In this study, we developed a workflow combining patient-specific three-dimensional reconstruction, thoracoscopic placement of temporary radiopaque anchors on the visceral pleural surface, and intraoperative CT verification of the anchor–nodule relationship. This retrospective study evaluated its technical feasibility and localization performance in selected patients and descriptively compared its outcomes with those of contemporaneous conventional CT-guided hookwire localization. Because localization strategy and resection technique differed concurrently between the cohorts, the comparison was considered exploratory and was not intended to establish comparative superiority.

## Methods

### Patient selection and data collection

This retrospective study included a consecutive cohort of 115 patients who underwent intraoperative model-guided anchor-based non-percutaneous localization or conventional preoperative CT-guided puncture localization and resection of small pulmonary nodules at our center between January 1 and December 31, 2024. All patients were scheduled for resection based on clinical indications, including suspicion of malignancy or radiologically indeterminate nodules.

Patients were eligible for the intraoperative-localization group if they met all of the following general criteria: (1) age ≥18 years; (2) one or more pulmonary nodules identified on preoperative CT; and (3) candidacy for VATS as determined by multidisciplinary evaluation. In addition, at least one of the following localization-specific criteria was required: (4) a ground-glass-dominant nodule located deep within the lung parenchyma, defined as a distance >10 mm from the geometric center of the nodule to the nearest visceral pleura; (5) absence of a suitable transthoracic puncture trajectory because of intervening anatomical structures or proximity to the mediastinum, diaphragm, or interlobar fissure; (6) multiple nodules for which repeated percutaneous punctures were considered undesirable; or (7) a documented patient preference for intraoperative localization after discussion of the available localization strategies, their potential benefits and risks, and the additional requirements of the hybrid-operating-room workflow. Criteria 4–7 were alternative rather than cumulative. Of the 24 patients, seven underwent intraoperative localization based on documented preference, without meeting criteria 4–6. Inclusion criteria for the conventional group were: (1) age ≥18 years, (2) no more than two pulmonary nodules on preoperative CT, (3) VATS candidacy, and (4) deemed localizable by the radiologist. Patients with significant comorbidities that precluded surgery or required emergent intervention were excluded. The institutional ethics committee approved this retrospective study (Approval No. 2025R0353-0002). The committee also confirmed that investigator assembly and temporary intraoperative use of radiopaque barium-thread markers prepared from commercially available sterile X-ray-detectable surgical gauze were permissible under institutional practice, provided that a standardized and reproducible accountability procedure was followed to prevent retention, as the markers were used only for temporary intraoperative localization and were not intended for permanent implantation.

Patient demographic and clinical data, including age, sex, smoking history, and comorbidities, were collected retrospectively from medical records. Nodule characteristics (size, location, and nodule depth, defined as the distance from the geometric center of the nodule to the nearest visceral pleura) were recorded from preoperative CT scans. Procedural details, including localization time, intraoperative findings, and complications, were extracted from the available records.

The localization technique utilized preoperative CT-based 3D reconstruction of the lung using Mimics software (Materialise NV, Leuven, Belgium). These models supported surgical planning and were used intraoperatively in a hybrid operating room equipped with advanced imaging. Localization was achieved through strategic placement of barium anchors, with accuracy confirmed during the procedure.

Postoperative outcomes assessed included localization success, resection margins, localization and operative times, and intra- or postoperative complications. Pneumothorax was defined as the presence of air in the pleural space separating the parietal and visceral pleurae. Pulmonary hemorrhage was defined as new ground-glass opacity in the affected parenchyma, per Fleischner Society criteria (14). Direct nodule puncture was defined as spatial superimposition of the needle and nodule on CT, and anchor dislodgement was defined as intraoperative visualization of the marker in the pleural space. Patients were monitored for 30 days postoperatively to identify localization-related adverse events.

### Methods of the intraoperative approach

The intraoperative localization workflow combined model-guided non-percutaneous initial anchor placement with a single CT-based assessment of the spatial relationship between the anchors and the target nodule. The CT findings were used to guide the direction and extent of resection. Although the anchors could theoretically be repositioned, neither physical repositioning of the anchors nor repeat CT verification was performed in this cohort. The intraoperative localization and nodule resection using the CONSERVO technique were performed by two experienced, licensed thoracic surgeons: one as chief surgeon (JL) and one as first assistant (XNZ); both have completed over 1,000 cases of uniportal thoracoscopic pulmonary excision and have an annual volume of over 200 cases. The steps were as follows:

#### Step 1: generation of the 3D model

Preoperatively, high-resolution chest CT scans (120 kVp, 35 mA, 0.625 mm slice thickness) were acquired for each patient using a SOMATOM Definition AS scanner (Siemens Medical Solutions USA, Inc., Pennsylvania, US).

The images acquired by the preoperative scan were processed by an experienced thoracic surgeon using Mimics software (Materialise NV, Leuven, Belgium) to create a 3D model of the lung that included the targeted nodule, parenchyma, pleura, and adjacent structures, with original CT slices incorporated (**Figure 1**). In the operating room, the model could be rotated, zoomed, and reoriented to approximate the actual lung position. The model was used as an aid to initial surgical planning.

**Figure 1:**
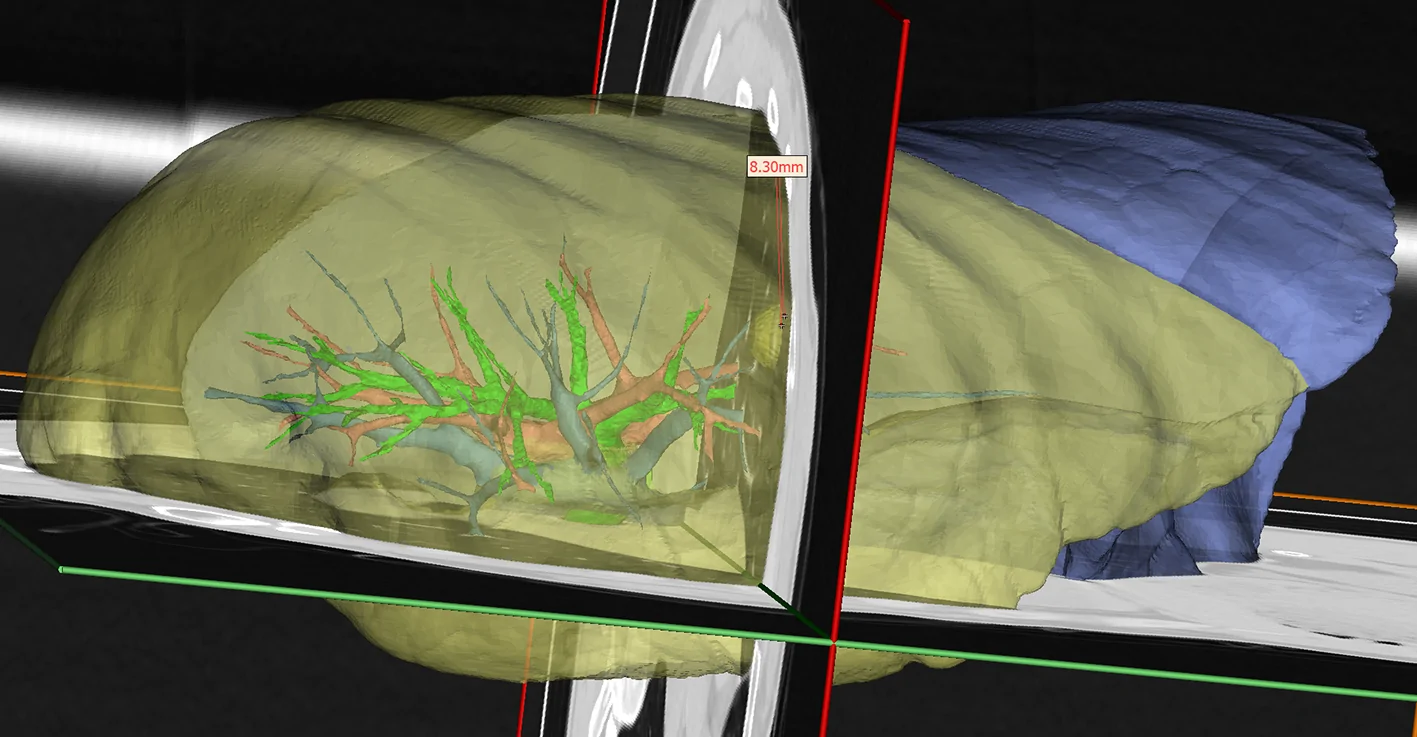
The 3D model that was used in the intraoperative localization approach. The 3D model generated by Mimics software. The contours of the left upper and lower lobes are displayed with corresponding CT images in multiple planes. These views were used to estimate the nodule’s pleural projection and plan initial anchor placement.

#### Step 2: crude localization using the 3D model

The 3D model was displayed intraoperatively to provide a preliminary map of the nodule’s location relative to anatomical landmarks. Measurement and marking were performed with the lung in a near-inflated state to account for heterogeneous deflation. Subpleural lymph nodes, accessory fissures, and impressions from adjacent structures served as landmarks when available. In lungs lacking these features, subpleural collecting pulmonary veins were used. When the pleura was heavily stained by sediment, intraoperative non-rigid curvature measurement aided mapping.

#### Step 3: assembly, placement, and evaluation of barium anchors

The pleural anchors were temporary, investigator-assembled intraoperative markers and were not intended for permanent implantation. Each anchor was prepared within the sterile operative field from a 3–4-mm segment of commercially supplied sterile X-ray-detectable gauze containing radiopaque barium thread and sterile 3–0 silk suture. To delineate the target zone, 1–5 barium anchors were positioned on the visceral pleural surface according to the preoperative 3D model. A round needle was passed superficially through the visceral pleura to draw each anchor into position, with the passage confined to the pleural layer and intended to avoid traversing the underlying lung parenchyma (**Figure 2a**). The anchors were secured without knotting and remained in position through friction at the pleural passage site (**Figure 2b**). Placement was guided by the model to cover the anticipated pleural projection of the nodule. A multiple-anchor strategy was often used to delineate the planned resection margin, providing redundancy against minor localization errors.

**Figure 2:**
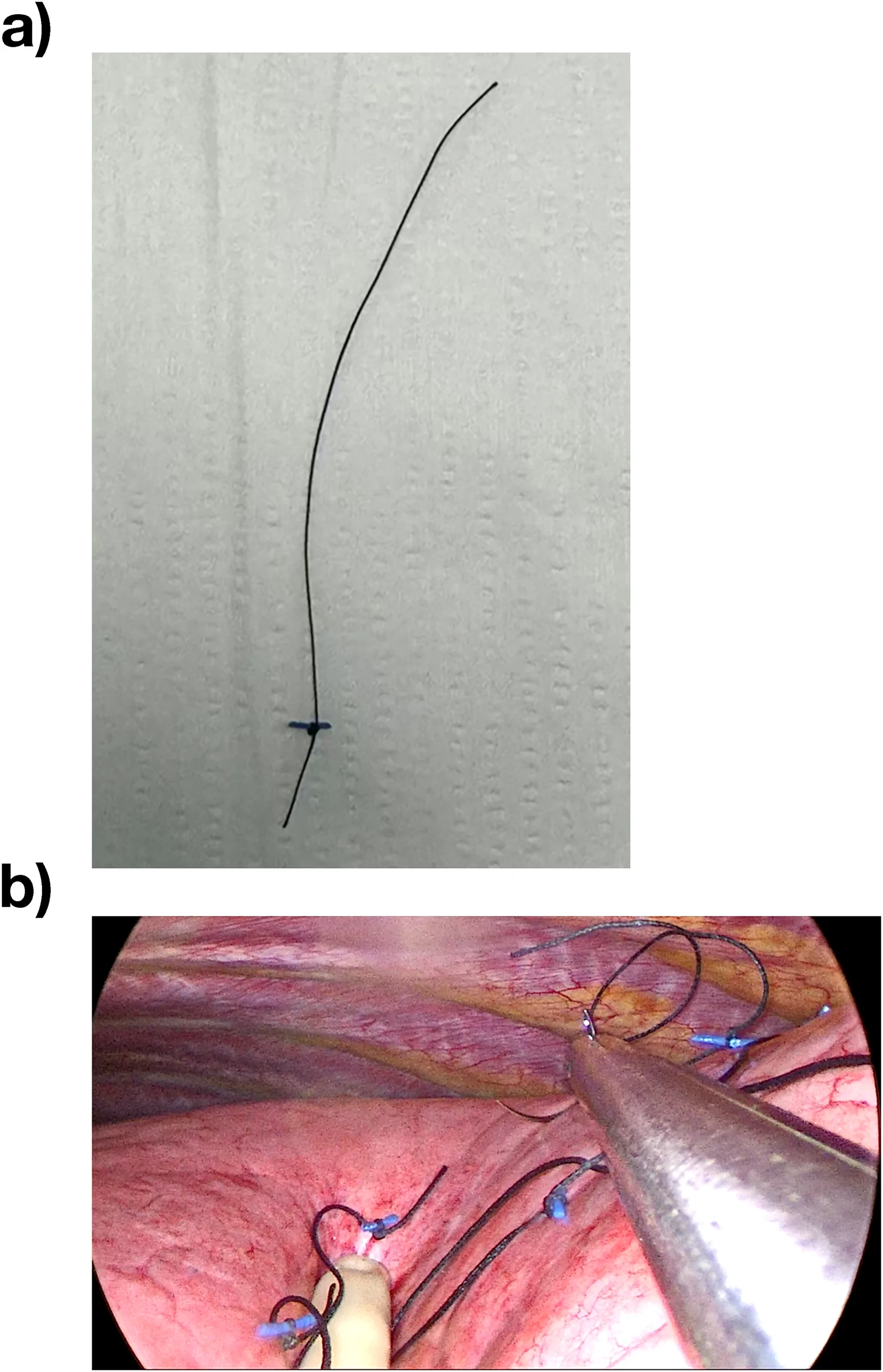
The anchor for intraoperative localization and its usage. **(a)** The structure of the anchor. The anchor we used was made from a short segment of barium thread obtained from the X-ray-detectable gauge, with the 3–0 silk thread securing it. This anchor was to be led with a round needle through the visceral pleura at the desired localization site. **(b)** The situated anchor on the visceral pleura. This figure shows anchor placement with the lung in a near-inflated state. A round needle was used to pass the anchor through the visceral pleura without knotting. The anchor remained in position during the subsequent scan in the illustrated case.

A standardized marker-accountability procedure, analogous to the institutional surgical-gauze count, was followed. The number and configuration of anchors required for each target were estimated preoperatively from the reconstructed model. The corresponding number of anchors was then assembled within the sterile field before placement. Anchor accountability followed the standardized count procedure described above, with the numbers prepared, placed, and retrieved, and the reconciliations completed before closure. During specimen retrieval, the operator and first assistant confirmed marker removal under thoracoscopic visualization. The scrub and circulating nurses reconciled the retrieved-marker count with the number prepared after specimen retrieval and again before incision closure. Markers located within the resection field were removed with the specimen; any marker outside the retrieved specimen was removed separately. Complete count reconciliation was achieved before closure in every case.

#### Step 4: intraoperative CT scan for anchor-nodule relationship

After anchor placement, an intraoperative CT scan (identical parameters: 120 kVp, 35 mA, 0.625 mm slice thickness, SOMATOM Definition AS) was performed in the hybrid operating room (**Figure 3a**). During the scan, the bilateral lungs were ventilated using a double-lumen endotracheal tube on a tidal volume of 10 mL/kg based on actual body weight of the patient and positive end-expiratory pressure of 8 cmH_2_O to restore the operative lung to a near-inflated state, under continuous monitoring and vital sign surveillance from the control room. Reconstructed images were used to assess spatial relationships between the anchor and the nodule. On-site 3D reconstruction from the intraoperative CT was performed when required (**Figure 3b**). After anchor placement, one intraoperative CT imaging episode was performed to assess the anchor–nodule relationship. When the initial CT demonstrated an offset but provided sufficient spatial information, the direction or extent of resection was modified accordingly without repositioning the anchors or obtaining repeat CT. When marker guidance remained insufficient, the predefined salvage strategy of thoracoscopic palpation followed, if unsuccessful, by anatomical segmentectomy was used.

**Figure 3:**
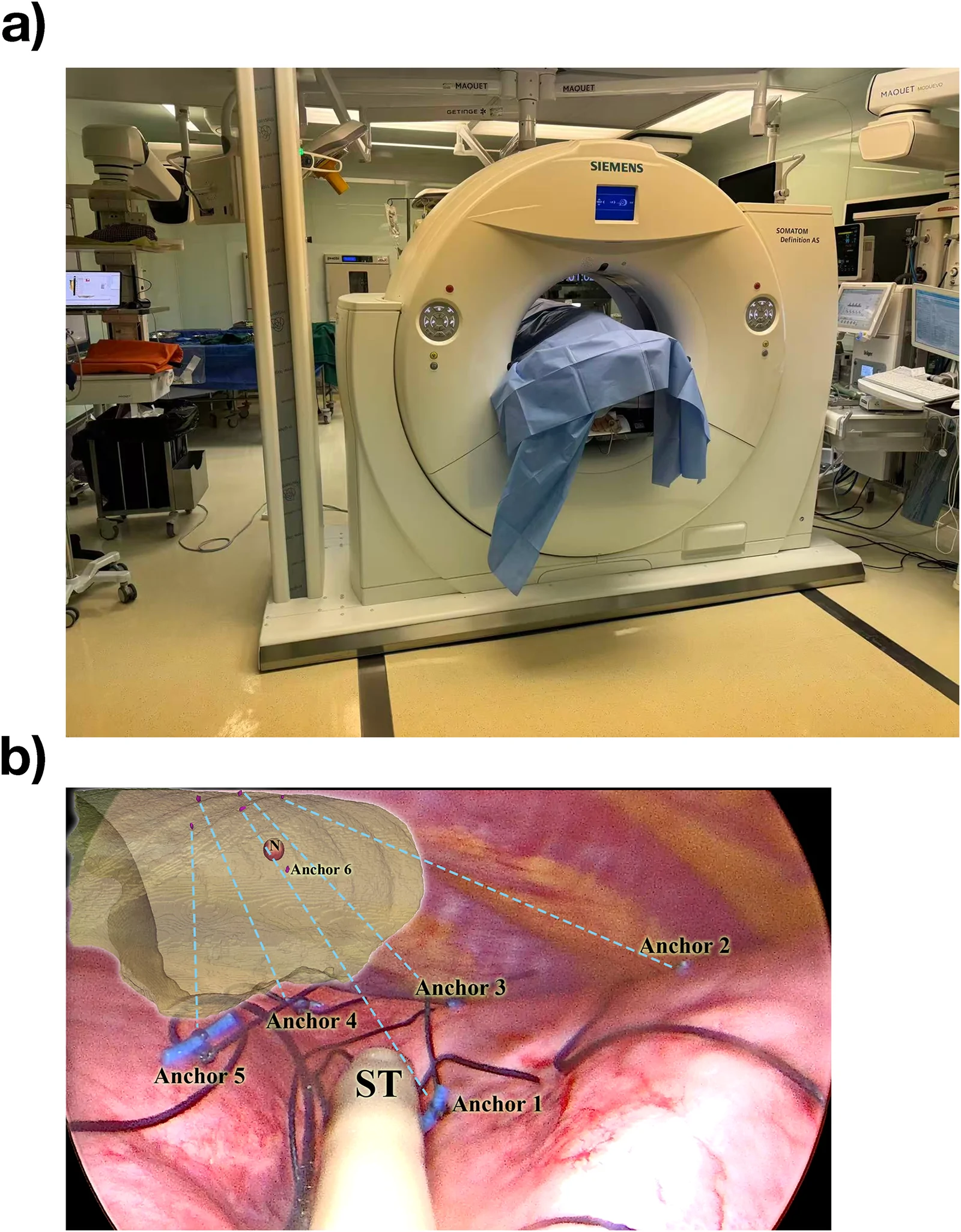
The intraoperative scan and the intraoperative 3D model with the anchor-labeled lung. **(a)** The intraoperative scanning. This figure presents the intraoperative scanning process. An additional sterile sheet was used to protect the operative field during CT positioning and image acquisition. Surgery resumed after completion of the scan and removal of the protective sheet. **(b)** The 3D model generated using the intraoperative scan and the anchor-labeled lung. This figure shows the right lower lobe and the 3D model generated from the intraoperative CT scan. The light-yellow colored 3D model of the right lower lobe was shown at the upper left corner. On it lay purple-colored anchors, linked with their respective actual positions by the cyan dotted line. The nodule’s location was then inferred according to the layout of anchors, helping determine the range of resection.

Radiation dose reports were reviewed for each operative scan in the patient protocol. The number of topograms and thoracic CT acquisitions, phantom designation, volume CT dose index (CTDIvol), and dose–length product (DLP) were extracted. Total DLP was obtained from the scanner-generated examination report. Approximate effective dose was calculated as total DLP multiplied by an adult-chest conversion coefficient of 0.014 mSv/(mGy·cm). Because effective dose is a population-based radiation-risk metric rather than an individually measured absorbed dose, the resulting values were interpreted as estimates. The clinical team comprised two surgeons, two nurses, and one anesthesiologist. Before each CT acquisition, all five personnel left the scanning area and remained in the hybrid operating room’s integrated shielded control area behind the fixed structural radiation barrier. No clinical personnel remained beside the patient during X-ray exposure. The control area and its structural shielding had passed the institution’s radiation-safety acceptance testing before clinical use. Physiological monitoring continued from the protected control area during image acquisition. Procedure-specific personnel dosimetry was not collected.

#### Step 5: resection and margin assessment

The area outlined by the anchors was resected according to the intraoperative CT and reconstructed model (typically requiring less than 10 minutes). Diathermal cutting in the partially deflated lung was used to improve correspondence between the planned and intraoperative geometry and to obtain the intended margin (15). The specimen was examined grossly for margin status, with additional resection performed if needed. Exposed parenchyma was closed with continuous 3–0 PDS II suture, followed by a water-seal test. A 20 Fr chest tube was placed, and the incision was closed in layers.

### Methods of the conventional approach

The conventional approach to localization and resection of small pulmonary nodules was performed in accordance with current guidelines (16). On the morning of surgery, patients underwent preoperative CT-guided hook-wire localization under local anesthesia in the radiology suite. After localization, patients returned to the ward to await operation. Intraoperatively, the resection area was planned based on the spatial relationship between the hook wire and the nodule on preoperative CT images. Resection was performed with staplers, and the specimen was grossly examined to confirm adequate margins. As in the intraoperative group, a water-seal test was conducted to detect significant air leakage, followed by layered closure of the incision after placement of a 20 Fr chest tube.

### Nodule localization accuracy assessment

Localization accuracy was assessed at the nodule level using postoperative review of the localization CT images. For conventional hookwire localization, the shortest three-dimensional distance between the hook tip and the nearest margin of the target nodule was measured. Localization was categorized as precise when this distance was ≤10 mm, helpful when it was >10 mm but ≤20 mm, and missed when it was >20 mm.

For intraoperative localization using a single anchor, the shortest distance between the center of the anchor and the nearest margin of the nodule’s pleural projection was measured using the intraoperative CT reconstruction. The same distance thresholds were applied: ≤10 mm was categorized as precise, >10 to ≤20 mm as helpful, and >20 mm as missed. When multiple anchors were used, localization was categorized as precise when the geometric center of the nodule’s pleural projection lay within the target region delineated by the anchors. When the projected center lay outside this region, localization was categorized as helpful if its shortest distance from the anchor-defined target region was ≤20 mm and as missed if this distance was >20 mm.

Localization categories were determined retrospectively by two thoracic surgeons (XNZ and JWL) through independent review of the CT images. Disagreements were resolved by consensus. Because the localization devices were visible on CT, reviewers could not be blinded to the localization approach.

When marker-based localization did not permit confident identification of the target nodule, a stepwise salvage strategy was used in both groups. Thoracoscopic palpation was performed first. If the target remained unidentifiable by palpation, the planned nonanatomic resection was abandoned, and segmentectomy encompassing the target lesion was performed according to its preoperative anatomical location. No additional intraoperative CT acquisition or repeat marker placement was performed in the cases included in this cohort.

### Procedural time definitions

Four procedural timestamps were available for each patient in the intraoperative group: skin incision, entry into the thoracic cavity, completion of intraoperative localization, and completion of incision closure. Three time measures were derived. Total operative time was defined as the interval from skin incision to completion of incision closure. In the intraoperative group, localization time was defined as the interval from entry into the thoracic cavity to final CT confirmation of the anchor-nodule relationship. Non-localization operative time was calculated by subtracting localization time from total operative time. For the linear regression analysis, the localization time was calculated on a normalized time per nodule using the following equation:


$$\text{Localization time per nodule}\;=\frac{\text{total licalization time for the patient}}{\text{number of nodules localized}}$$

In the conventional group, preoperative hookwire localization was performed separately in the radiology suite; therefore, operative time was defined as the time from skin incision to completion of incision closure and did not include the localization component.

### Postoperative care and follow-up

Postoperative management included prophylactic antibiotics, patient-controlled intravenous analgesia, and anticoagulant therapy. A chest radiograph was obtained on postoperative day 1 to assess lung re-expansion. Routine laboratory tests (complete blood count, biochemistry, and coagulation profile) were performed every other day starting on postoperative day 1. The chest tube was removed when all the following criteria were met: (1) full lung re-expansion on chest radiograph with stable water-seal level during coughing or speaking, (2) drainage volume <200 mL in the preceding 24 hours, and (3) serous fluid color. Patients were typically discharged the day after chest tube removal if clinically stable.

Postoperative surveillance was scheduled according to pathological diagnosis. Patients with malignant pulmonary lesions underwent non-contrast chest CT, ultrasonography of the abdomen and superficial lymph-node regions, and measurement of serum tumor markers (CEA, CA19-9, SCC, CYFRA21-1, NSE, and ProGRP) every 3 months. Patients with benign lesions underwent non-contrast chest CT every 6 months. The data cutoff for follow-up was July 1, 2026.

For patients with malignant lesions, recurrence-free survival was defined as the interval from surgery to the first documented disease recurrence. Overall survival was defined as the interval from the date of surgery to death from any cause. Follow-up times were calculated in months. Patients without recurrence were censored for recurrence-free survival at the date of their last documented disease assessment or July 1, 2026, whichever occurred first. Patients who remained alive were censored for overall survival at the date they were last known to be alive or July 1, 2026, whichever occurred first.

### Statistical analysis

Data were analyzed to describe the safety and localization outcomes of the two approaches, with between-group comparisons considered exploratory. Statistical analyses were performed using STATA/MP version 15.1 (StataCorp LLC, College Station, TX, USA), R version 4.5.1 (R Foundation for Statistical Computing, Vienna, Austria) (17). Baseline characteristics (age, sex, smoking history, nodule size, and location) were summarized with descriptive statistics. Continuous variables are reported as mean ± standard deviation (SD) or median with interquartile range (IQR), according to their distribution. Categorical variables are presented as frequencies and percentages. An exploratory linear regression was used to examine the association between procedural experience and localization time per nodule, with additional analysis of the first and last quartiles of intraoperatively localized patients. Procedural experience was represented by days since the first case. Because of the limited cohort size, no proficiency threshold or performance plateau was prespecified. A two-tailed p-value < 0.05 was considered statistically significant.

## Results

### Overview

A total of 115 patients underwent nodule localization and resection. Of these, 24 patients received intraoperative localization, and 91 received preoperative CT-guided puncture localization and resection. Recorded baseline characteristics are presented in **Table 1**. Comorbidities were more frequent in the conventional group (51.65% vs. 29.17%, p = 0.05).

**Table 1:** The baseline characteristics of patients.

Variables	intraoperative localization n = 24	Conventional n = 91	p value
Age (year)	59 (49-64)	59 (50-64)	0.910
Sex			0.179
Male	10 (41.67%)	25 (27.47%)	
Female	14 (58.33%)	66 (72.53%)	
Smoking (pack-year)	0 (0-0)	0 (0-0)	0.573
History of malignancy			1.000
Yes	1 (4.17%)	4 (4.40%)	
No	23 (95.83%)	87 (95.60%)	
Comorbidity			0.050
Yes	7 (29.17%)	47 (51.65%)	
No	17 (70.83%)	44 (48.35%)	
Location of the nodules			0.614
RUL	10 (41.67%)	32 (35.16%)	
RML	0 (0%)	3 (3.3%)	
RLL	3 (12.50%)	17 (18.68%)	
LUL	7 (29.17%)	13 (14.29%)	
LLL	3 (12.50%)	19 (20.88%)	
RUL and RLL	1 (4.17%)	2 (2.2%)	
RUL and RML	0 (0%)	2 (2.2%)	
LUL and LLL	0 (0%)	3 (3.3%)	

RUL, right upper lobe; RML, right middle lobe; RLL, right lower lobe; LUL, left upper lobe; LLL, left lower lobe.

### Localization-specific outcomes

Thirty nodules were localized using the intraoperative approach and 99 using the preoperative approach. The nodules in the preoperative group were deeper (8.96 [4.88-13.25] vs. 4.99 [2.34-12.30], p = 0.041). Localization accuracy was comparable between groups (intraoperative vs. preoperative: precise 50% vs. 49.49%; helpful 43.33% vs. 42.42%; missed 6.67% vs. 8.08%; p = 1.000). In the intraoperative localization group, each patient received one scan during the operation. All 10 nodules categorized as missed were ultimately managed using the stepwise salvage strategy. In the intraoperative group, one of the two missed nodules was successfully localized by thoracoscopic palpation, whereas the other required anatomical segmentectomy. In the conventional group, six of the eight missed nodules were successfully localized by palpation, and two required anatomical segmentectomy.

A total of 62 anchors were used for 30 nodules in 24 patients. The median number of anchors per case was 2.5 (IQR, 1–4; range, 1–5). One anchor was used in 9 cases, two in 3 cases, three in 2 cases, four in 9 cases, and five in 1 case. The number of anchors prepared matched the number placed and retrieved in every case, with no count discrepancy or retained anchor recorded.

No repeat intraoperative CT acquisition or repeat marker placement was performed. Median CTDIvol across the 24 thoracic acquisitions was 6.87 mGy (IQR, 5.76–7.94; range, 5.23–10.88). Median total DLP per imaging episode was 198.5 mGy·cm (IQR, 161.3–230.8; range, 119–387), corresponding to an estimated median effective dose of 2.78 mSv (IQR, 2.26–3.23; range, 1.67–5.42). Intraparenchymal hemorrhage was observed in 0 of 30 nodules (0.0%) in the intraoperative group and 16 of 99 nodules (16.2%) in the conventional group (p = 0.022). Pneumothorax occurred in 0 of 24 patients (0.0%) and 29 of 91 patients (31.9%), respectively (p < 0.001). Direct nodule puncture was recorded for 0 of 30 nodules (0.0%) in the intraoperative group and 10 of 99 nodules (10.1%) in the conventional group (p = 0.115). Marker dislodgement was recorded for 0 of 30 nodules (0.0%) and 1 of 99 nodules (1.0%), respectively (p = 1.000), although the between-group differences were not statistically significant. More nodules per patient were localized in the intraoperative group (median [IQR], 1 [1-2] vs. 1 [1-1], p = 0.004). In an exploratory patient-level regression, localization time, normalized by the number of nodules, decreased with days since the first procedure (β = −0.156 min/day, 95% CI, −0.294 to −0.019; p = 0.028; R² = 0.201, **Figure 4**). However, descriptive comparison of the first and final quartiles did not demonstrate a consistent reduction in normalized localization time. Median patient-level localization time was 39.2 minutes (IQR, 27.6–56.6) among the first six cases and 35.7 minutes (IQR, 33.9–37.9) among the final six. Median localization time normalized per nodule was 31.0 minutes (IQR, 14.7–56.6) and 34.6 minutes (IQR, 31.3–37.9), respectively. These findings suggest reduced variability over time but do not establish a proficiency threshold or a monotonic learning curve. This analysis describes a temporal trend and does not establish a proficiency threshold. Pathological findings did not differ significantly between groups (**Table 2**).

**Figure 4:**
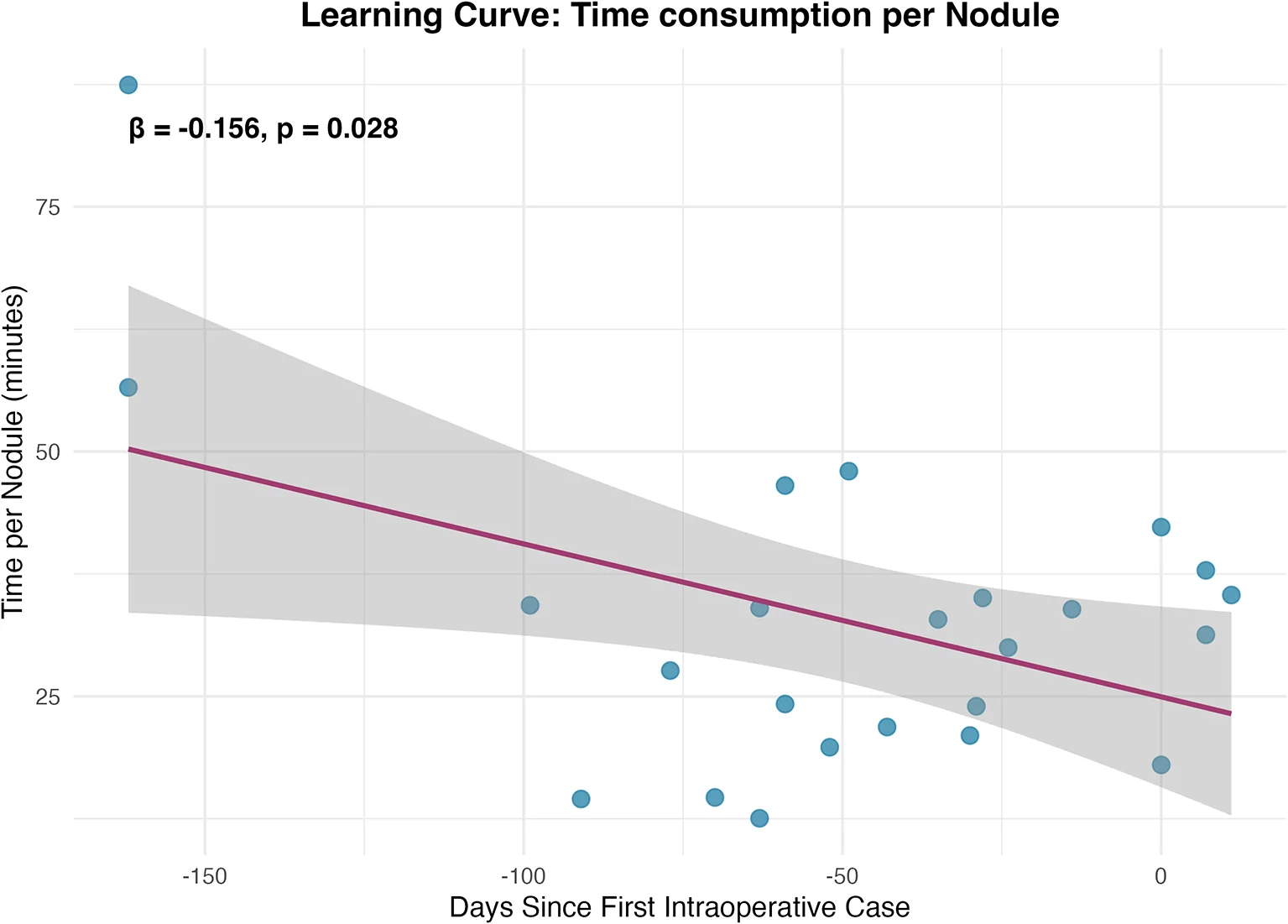
Association between procedural date and localization time per nodule. The scatter plot shows the relationship between the number of days since the first intraoperative case and the normalized localization time per nodule. The solid line represents the linear regression fit, and the shaded area represents the 95% confidence interval (β = −0.156 min/day, p = 0.028). This exploratory analysis does not define a proficiency threshold.

**Table 2:** Results of the nodule localization.

Variables	Intraoperative localization	Conventional	p value
Patient-level outcome	n = 24 patients	n = 91 patients	
Post-localization Pneumothorax*			<0.001
Yes	0 (0%)	29 (31.87%)	
No	24 (100%)	62 (68.13%)	
Nodule-level outcome	n = 30 nodules	n = 99 nodules	
Depth of the nodules† (mm)	4.99 (2.34-12.30)	8.96 (4.88-13.25)	0.041
Localization results			1.000
Precise	15 (50%)	49 (49.49%)	
Helpful	13 (43.33%)	42 (42.42%)	
Missed	2 (6.67%)	8 (8.08%)	
Intraparenchymal Hemorrhage			0.022
Yes	0 (0%)	16 (16.16%)	
No	30 (100%)	83 (83.84%)	
Direct Nodule Puncture			0.115
Yes	0 (0%)	10 (10.10%)	
No	30 (100%)	89 (89.90%)	
Marker Dislodgement			1.000
Yes	0 (0%)	1 (1.01%)	
No	30 (100%)	98 (98.99%)	
Resectional Margin			1.000
Positive	0 (0%)	1 (1.01%)	
Clear	30 (100%)	98 (98.99%)	
Malignant or Benign			0.451
Benign	1 (3.33%)	9 (9.09%)	
Malignant	29 (96.67%)	90 (90.91%)	
Pathological types‡			0.096
AAH	4 (13.79%)	10 (11.11%)	
AIS	6 (20.69%)	39 (43.33%)	
MIA	11 (37.93%)	27 (30.00%)	
IAC	6 (20.69%)	12 (13.33%)	
Others	2 (6.90%)	2 (2.22%)	
Tumor diameter (mm)	7 (5-9)	7 (5-8)	0.799

AAH, atypical adenomatous hyperplasia; AIS, adenocarcinoma *in situ*; MIA, minimally invasive adenocarcinoma; IAC, invasive adenocarcinoma;.

*Pneumothorax was analyzed at the patient level because it represented a complication of the overall localization episode; no event was counted more than once in patients undergoing localization of multiple nodules. All other variables in this table were analyzed at the nodule level.

†The depth of the nodules was defined as the distance from the geometric center of the nodule to the nearest visceral pleura.

‡Pathological subtype was summarized among malignant nodules only, comprising 29 nodules in the intraoperative group and 90 nodules in the conventional group. Benign nodules were excluded, and percentages within each group were calculated using the corresponding number of malignant nodules as the denominator.

### Resection and perioperative outcomes of the combined procedural strategies

The extent of pulmonary resection was similar between groups (p=0.914). However, the parenchymal division techniques differed completely: all nonanatomic resections in the intraoperative-localization group were performed using CONSERVO electroresection, whereas those in the conventional-localization group were stapler-based. Therefore, the following comparisons represent outcomes of the combined procedural strategies rather than effects attributable independently to either localization or resection technique.

All patients in the intraoperative localization group achieved complete resection with negative margins; in the preoperative group, one patient had incomplete resection and subsequently underwent completion lobectomy. As of July 1, 2026, no patient was lost to follow-up; the median follow-up time was 24 months (IQR, 22–27 months, range, 18–30 months); no recurrence or death was documented during follow-up in either group. Median total operative time was longer in the intraoperative-localization/CONSERVO group than in the conventional group (192.0 minutes [IQR, 134.5–271.5] vs. 75.0 minutes [IQR, 56.0–119.0], p < 0.001). Median intraoperative localization time was 35.7 minutes per patient (IQR, 28.8–47.3). After subtraction of localization time, median non-localization operative time remained longer in the intraoperative group (147.3 minutes [IQR, 102.6–231.0] vs. 75.0 minutes [IQR, 56.0–119.0], p < 0.001). The intraoperative group had a shorter mean incision length (20.88 ± 3.35 mm vs. 30.81 ± 5.94 mm, p < 0.001), but higher intraoperative blood loss (20 mL [10-30] vs. 10 mL [10-20], p = 0.029), longer chest tube duration (3 days [2-4] vs. 2 days [2-3], p = 0.013), and greater total drainage volume in the first 3 postoperative days (210 mL [117-270] vs. 130 mL [77-205], p = 0.040) (**Table 3**). Because localization strategy and resection technique differed between groups, these perioperative differences cannot be attributed independently to either component.

**Table 3:** Resection and perioperative outcomes of the combined procedural strategies.

Variables	Intraoperative localization n = 24	Conventional n = 91	p value
Extent of pulmonary resection*			0.914
Nonanatomic sublobar resection	20 (83.33%)	76 (83.52%)	
Anatomical segmentectomy	2 (8.33%)	9 (9.89%)	
Combined nonanatomic sublobar resection and segmentectomy	2 (8.33%)	5 (5.49%)	
Combined nonanatomic sublobar resection and lobectomy	0 (0%)	1 (1.1%)	
Length of incision (mm)	20.88 ± 3.35	30.81 ± 5.94	<0.001
Nodule localized	1 (1-2)	1 (1-1)	0.004
Non-localization operative time† (minutes)	147 (102-230)	75 (56-119)	<0.001
Intraoperative bleeding (mL)	20 (10-30)	10 (10-20)	0.029
Time of tube placement (days)	3 (2-4)	2 (2-3)	0.013
Drainage volume within 3 days (mL)	210 (117-270)	130 (77-205)	0.040

All patients in the intraoperative-localization group underwent CONSERVO electroresection, whereas patients in the conventional-localization group underwent stapler-based resection. Consequently, the reported differences cannot be attributed independently to localization or resection technique.

*Extent of pulmonary resection was categorized independently of the parenchymal division technique. “Nonanatomic sublobar resection” included conventional stapled wedge resection in the conventional group and conformal nonanatomic sublobar resection performed using the CONSERVO technique in the intraoperative group. Anatomical segmentectomy and lobectomy were classified according to the anatomical extent of resection. The parenchymal resection technique was recorded separately as CONSERVO electroresection or stapler-based resection.

†Total operative time was defined as skin incision to completion of incision closure. Intraoperative localization time was defined as the time from thoracic-cavity entry to completion of localization. Non-localization operative time was calculated by subtracting localization time from total operative time and therefore included surgical access, resection, and closure. In the conventional group, hookwire localization was performed in the radiology suite before surgery; therefore, total and non-localization operative times were identical and excluded preoperative localization.

## Discussion

In this exploratory retrospective study, intraoperative model-guided pleural anchor placement with CT verification produced localization-category distributions similar to those observed with conventional hookwire localization. No pneumothorax or pulmonary hemorrhage attributable to localization was observed in the intraoperative cohort. An exploratory regression suggested a reduction in localization time per nodule over the study period, although the small cohort and variability among early cases preclude definition of a learning curve or proficiency threshold. Overall, these findings support the technical feasibility of the workflow in selected patients but do not establish superiority or equivalence relative to established localization methods.

A persistent methodological challenge is the registration of preoperative CT anatomy to the intraoperative lung. Diagnostic CT is generally acquired with the patient supine and the lungs fully inflated, whereas localization and resection may be performed in the lateral decubitus position under altered ventilation. Changes in position, inflation, gravitational deformation, pleural adhesions, and regional differences in parenchymal collapse may alter both the apparent pleural projection of a nodule and its relationship to surface landmarks (11, 12). Consequently, a surface position estimated from preoperative imaging may not correspond exactly to the nodule’s intraoperative projection.

Several methods are available for the localization of small pulmonary nodules, including hookwire, microcoil, liquid-marker injection, and bronchoscopic or fluorescence-guided approaches. CT-guided percutaneous indocyanine green (ICG) localization has achieved high technical success and is increasingly used in centers equipped with near-infrared thoracoscopy. Retrospective comparisons suggest that ICG may reduce localization time, procedural discomfort, wire dislodgement, and overall complication rates compared with hookwire, although reductions in pneumothorax and pulmonary hemorrhage have not been consistent across individual studies. Percutaneous ICG nevertheless requires transthoracic needle access, whereas bronchoscopic ICG requires appropriate bronchial access, navigation equipment, and specialized expertise (18). Therefore, localization strategy should be individualized according to lesion characteristics, available expertise, equipment, and institutional workflow.

Intraoperative pulmonary nodule localization within a hybrid operating room is not itself novel. Previous approaches have included intraoperative CT-guided percutaneous placement of hookwires, coils, or dyes; CT-assisted thoracoscopic placement of visceral-pleural titanium clips (13); virtual imaging-assisted pleural marking (19); and patient-specific three-dimensional navigational templates (9). Collectively, these techniques seek to translate preoperative imaging information into an intraoperative landmark with clinically acceptable accuracy and procedural burden. Nevertheless, individual approaches retain limitations related to transthoracic puncture, dependence on estimated surface projection, specialized equipment, or repeated imaging. For example, the visceral-pleural titanium-clip technique reported by Yu et al. required a median of three intraoperative CT acquisitions (range, 2–4) to achieve satisfactory localization, indicating a potential trade-off between iterative spatial confirmation and imaging burden (13).

Therefore, localization strategies differ in their route of access, timing, resource requirements, and suitability for individual lesions. Percutaneous hookwire, microcoil, and liquid-marker techniques are widely available and generally achieve high technical success but require transthoracic access and may cause pneumothorax, pulmonary hemorrhage, discomfort, marker migration, or dye diffusion. Bronchoscopic approaches avoid transthoracic puncture and may be advantageous for selected deep or multifocal lesions, but their performance depends on bronchial access, navigation equipment, and specialized expertise. Anatomy- and model-based approaches avoid percutaneous localization but may be affected by lung deformation between preoperative imaging and surgery. **Supplementary Table S1** summarizes these considerations and positions the present workflow as a complementary option for selected patients rather than a replacement for established localization methods.

The present approach combines patient-specific preoperative three-dimensional modeling, thoracoscopic placement of one or more temporary radiopaque anchors on the visceral pleural surface, and intraoperative CT verification of the anchor–nodule relationship. Multiple anchors were used when appropriate to delineate a target region rather than a single estimated projection point. This combination was intended to preserve the planning value of preoperative imaging while providing intraoperative spatial evaluation without intentional transthoracic needle traversal of the lung parenchyma. The present study evaluates the feasibility, localization performance, and practical limitations of this workflow; it does not establish its superiority over other hybrid operating-room or image-guided localization methods.

The barium anchors used in this study were investigator-assembled temporary markers, with institutional ethics committee approval and a reproducible standard of operation to prevent retention or fragmented anchors. Because the anchors were not grasped, stretched, or subjected to direct traction after placement, fragmentation was considered unlikely, and no visible fragmentation or count discrepancy occurred in this cohort. Nevertheless, the construct was not designed or validated as a dedicated localization marker, and fragmentation or retention of radiopaque material remains a theoretical risk. Strict visual retrieval and count reconciliation are therefore required, and future development should use a standardized, purpose-designed marker with formal biocompatibility and mechanical testing.

Localization quality was categorized as precise, helpful, or missed to reflect the extent to which the marker configuration informed resection planning. The multi-anchor strategy was intended to provide spatial information beyond a single pleural point and was used for both localization and margin planning. The intraoperative group included more nodules localized per patient than the conventional group (median [IQR], 1 [1-2] vs. 1 [1-1], p = 0.004), suggesting that multifocal cases may be a relevant population for further study.

When CT demonstrated imperfect anchor-nodule alignment, the operative plan or anchors could be adjusted without additional CT acquisition; however, in this cohort, intraoperative CT served as a single spatial-verification step rather than as part of an iterative scan–adjust–rescan workflow. Although the anchor design theoretically permits repositioning, this function was not used or evaluated. For helpful localizations, the CT-demonstrated anchor–nodule relationship was used to modify the resection plan without repeat imaging. The two missed localizations were managed through palpation or anatomical segmentectomy. Consequently, the present results support a CT-based interpretation of the initial anchor configuration but do not establish the safety, accuracy, or practical value of physically adjusting the anchors.

Contrary to an anticipated association between intraoperative localization and greater nodule depth, nodules in the conventional group had a greater median pleura-to-center distance. This finding indicates that selection of the intraoperative approach was not determined solely by depth. Percutaneous accessibility also depends on the available needle trajectory and the lesion’s relationship to the mediastinum, diaphragm, fissures, scapula, and adjacent bronchovascular structures; therefore, a relatively superficial nodule may still be unsuitable for safe percutaneous localization. Nevertheless, depth does not capture all dimensions of anatomical complexity, and indication-based allocation, multifocality, and other unmeasured factors may still have confounded the between-group comparison.

During the study period, hookwire was the conventional preoperative localization method used at our center and consequently served as the contemporaneous comparator. Thus, the present results should not be interpreted as establishing superiority over ICG or other modern localization techniques, despite the clearly recorded and reproducible workflow of 3D-reconstructed model-guided intraoperative localization. Rather, the intraoperative approach may represent a complementary option for selected patients with anatomically unsuitable puncture trajectories, multiple nodules, or lesions unlikely to be localized by palpation.

No intrapulmonary hemorrhage, pneumothorax, direct nodule puncture, or anchor dislodgement was observed in the intraoperative cohort. Pneumothorax is a recognized complication of percutaneous preoperative localization, with reported rates varying across techniques and centers (20, 21). The rate in the conventional group in this study was 31.87%. Most events were minor and did not preclude subsequent surgery; nevertheless, they represent additional procedural morbidity and may require monitoring or intervention. Rare serious events have also been reported (22). From a population-based perspective, Iguchi et al. identified the transfissural approach and longer procedure time as independent predictors of post-procedural pneumothorax (23). This finding supports further investigation in patients with high-risk puncture trajectories.

Pulmonary hemorrhage is another recognized consequence of transthoracic needle access and can be graded according to the extent of ground-glass opacity (24). Hemorrhages observed after conventional localization in this series were grade 1 or 2, and no subsequent infection or other hemorrhage-related postoperative complication was recorded. Although these events were clinically limited, their absence in the intraoperative cohort is consistent with the non-percutaneous design of the technique. The clinical importance of this difference requires confirmation in a balanced prospective comparison.

Marker dislodgement can complicate localization and may necessitate a change in resection strategy or repeat localization. Reported hookwire dislodgement rates range from 3% to 8% (25–27), and remedial localization strategies have been described (28). No anchor dislodgement was observed in the intraoperative cohort, indicating a potentially lower risk of dislodgement of the intraoperative localization technique.

Systemic air embolism is a rare but potentially serious complication of percutaneous lung procedures. A rate of 0.5% has been reported in one localization cohort (29), and fatal cases have been described (6, 7). Proposed mechanisms include communication between airways and injured pulmonary veins or direct entry of air into the pulmonary venous circulation (30–32). By avoiding transthoracic needle access, the present technique avoids these specific needle-related mechanisms; however, the current sample was too small to assess rare-event risk or establish a comparative safety advantage.

Although exploratory regression suggested a decrease in localization time with procedural date, the first- and last-quartile medians did not show a consistent reduction in time normalized per nodule. The principal descriptive change was narrower dispersion in the final quartile, suggesting that workflow execution may have become more consistent. Given the small number of cases and possible changes in case mix, this observation is hypothesis-generating and does not define a learning curve or proficiency threshold.

The distribution of pulmonary resection extent was similar between groups; however, the parenchymal resection technique differed completely. Nonanatomic sublobar resections in the intraoperative group were performed using CONSERVO electroresection (15), whereas those in the conventional group were performed using staplers. Intraoperative localization time was recorded separately and excluded from the reported non-localization operative time; therefore, the observed 72-minute difference in this measure did not directly represent time spent on localization. Nevertheless, the perioperative findings should be interpreted at the level of the combined procedural strategies. The shorter incision observed in the intraoperative cohort is more plausibly related to the use of CONSERVO electroresection than to the localization method itself. Conversely, the longer non-localization operative time, greater blood loss, longer chest-tube duration, and greater early drainage may reflect the resection technique, case selection, multifocality, operator factors, or other unmeasured differences. Because localization and resection techniques were completely aligned within their respective groups, their independent contributions cannot be estimated from this study.

Lastly, cost and resource requirements should also be considered. The present workflow requires access to a hybrid operating room with intraoperative CT capability, preoperative three-dimensional model preparation, coordinated surgical, anesthesia, nursing, and imaging personnel, and additional operating-room occupation during localization and scanning. Establishing such infrastructure entails substantial capital and maintenance costs, and its use for localization may compete with other procedures requiring hybrid-operating-room access. The marginal cost may be lower at centers where the necessary infrastructure and personnel are already available, but this was not evaluated in the present study. Because the intraoperative localization procedure remained exploratory, patients were not charged a separate localization fee, whereas conventional preoperative hookwire localization generated a clinical charge. This difference reflects institutional billing practice and should not be interpreted as evidence that the intraoperative workflow is less costly or cost-effective. Formal economic evaluation should include capital allocation, personnel time, imaging, anesthesia duration, operating room occupancy, consumables, and patient-incurred costs.

This study has several limitations. First, it was a single-center retrospective study with a small intraoperative cohort, which limited its precision, generalizability, and ability to assess rare events. Second, treatment allocation was nonrandom and indication-based, creating selection bias and limiting causal interpretation of between-group differences. Patient referral and operator availability may also have influenced treatment allocation. The intraoperative localization workflow was performed by a dedicated surgical team with expertise in this technique, and patients considered for this approach were preferentially referred to that team. Accordingly, the study cohort may reflect both clinical indications and center-specific referral patterns, thereby limiting generalizability and introducing potential selection bias. Third, the workflow requires hybrid operating-room infrastructure, preoperative model preparation, multidisciplinary coordination, controlled ventilation during imaging, protection of the sterile field, radiation exposure, and additional operating-room occupation. These requirements restrict its accessibility and make routine use inappropriate in centers without the necessary infrastructure and experience. Additionally, solitary superficial nodules that can be reliably localized using simpler methods are unlikely to derive meaningful benefit. The potential application of this workflow is therefore limited to selected patients with multiple nodules, unsuitable percutaneous trajectories, poorly palpable lesions, or a need for immediate intraoperative evaluation. The feasibility and accuracy of physical anchor repositioning, followed by repeat CT verification, were not evaluated and require a prospective study. Furthermore, although preoperative 3D reconstruction guided initial anchor placement and intraoperative CT enabled evaluation, the study did not compare this workflow with direct surgeon estimation followed by CT. The independent contribution of 3D modeling to accuracy, scan frequency, and procedural time therefore remains uncertain. Additionally, although every patient underwent only one intraoperative CT acquisition and patient-specific CTDIvol and DLP were retrieved and analyzed, quantitative comparison with radiation exposure during conventional CT-guided localization was not performed. Although all personnel remained in the hybrid operating room’s tested shielded control area during acquisition, procedure-specific occupational dosimetry was not recorded. Future prospective studies should retain the scanner-generated radiation dose structured report for every acquisition and collect procedure-linked personnel dosimetry where appropriate. Lastly, localization strategy and resection technique were completely confounded, preventing separation of their effects on perioperative outcomes. Prospective multicenter studies using standardized eligibility criteria, contemporary comparators, explicit procedural time components, radiation reporting, and patient-reported outcomes are required.

## Conclusion

In this retrospective, indication-selected cohort, intraoperative model-guided anchor placement with CT verification was technically feasible and enabled non-percutaneous localization of selected pulmonary nodules. The observed localization and safety outcomes support further evaluation, particularly for multifocal or puncture-ineligible lesions in centers with hybrid operating-room capability. These findings do not establish comparative superiority, routine clinical utility, or broad generalizability.

## Data Availability

The raw data supporting the conclusions of this article will be made available by the authors, without undue reservation.
