# Supplementary material for: Intraoperative model-guided anchor placement with CT verification for non-percutaneous localization of small pulmonary nodules: a retrospective feasibility study
Source: Front Oncol. 2026 Oct 19;16:1941071. doi: 10.3389/fonc.2026.1941071 (PMC13724330; doi:10.3389/fonc.2026.1941071)
Supplement: Supplementary file 1 [file Table1.docx]

**Supplementary Table S1 Comparative characteristics of pulmonary nodule localization strategies**

| **Strategy** | **Timing and access route** | **Localization characteristics** | **Principal limitations and complications** | **Resource requirements** | **Potentially suitable situations** |
| --- | --- | --- | --- | --- | --- |
| CT-guided hookwire [S1,S2] | Usually preoperative; percutaneous transthoracic placement in a CT suite | Direct physical marker near the lesion; generally high reported technical success | Pain, pneumothorax, pulmonary hemorrhage, wire migration or dislodgement, and a waiting interval before surgery | CT suite and interventional expertise; hybrid OR not required | Solitary peripheral nodules with a safe transthoracic trajectory |
| CT-guided microcoil [S2,S3] | Usually preoperative; percutaneous transthoracic placement | Radiopaque marker with generally high reported technical success; may be deployed partly across the pleural surface | Pneumothorax, pulmonary hemorrhage, displacement, and radiation exposure; intraoperative fluoroscopy is required in some workflows | CT suite; fluoroscopy may be required; hybrid OR not routinely required | Small or deep nodules with an accessible puncture trajectory |
| Percutaneous liquid markers, including methylene blue, ICG, barium, and lipiodol [S4,S5] | Usually preoperative; can be performed intraoperatively under CT guidance | Visual, fluorescent, or radiopaque marking; technical performance depends on the material and protocol | Needle-related pneumothorax or hemorrhage; diffusion, spillage, or fading; material-specific adverse effects and imaging requirements | CT guidance; ICG requires near-infrared thoracoscopy; intraoperative placement requires hybrid-OR imaging | Peripheral nodules; ICG is useful when near-infrared imaging is available |
| Navigational or robotic bronchoscopic marking [S6] | Preoperative or intraoperative under anesthesia; transbronchial dye injection or marker placement | Avoids transthoracic puncture and may permit localization of multiple lesions during one bronchoscopic session | Dependent on an accessible bronchial route, navigation accuracy, equipment, and expertise; dye diffusion or marker migration may occur | Electromagnetic or robotic navigation; adjunct cone-beam CT may improve confirmation; hybrid OR not invariably required | Deep or multifocal lesions with an accessible bronchial route, particularly when transthoracic puncture is undesirable |
| Anatomy- or model-based localization, including virtual imaging and 3D-printed templates [S7,S8] | Intraoperative; uses preoperative CT-derived anatomical or pleural landmarks | Avoids percutaneous localization and estimates the lesion's pleural projection | Registration may be affected by patient position, inflation, pleural adhesions, and non-uniform lung deformation; preparation is method-specific | Segmentation/modeling expertise; printing or navigation resources may be required; hybrid OR generally not required | Peripheral lesions with reproducible anatomical or pleural landmarks |
| Thoracoscopic visceral-pleural clips with intraoperative CT [S9] | Intraoperative after thoracic entry; clips placed on the visceral pleura and assessed by CT | Provides a radiopaque pleural-surface landmark without percutaneous marker placement | May require iterative clip placement and CT acquisition; entails radiation exposure and hybrid-OR occupation | Hybrid OR with intraoperative CT and coordinated surgical, anesthesia, nursing, and imaging support | Selected peripheral nodules for which a surface marker can guide resection |
| Model-guided pleural-anchor placement with intraoperative CT verification (present workflow) | Intraoperative after thoracic entry; temporary anchors placed thoracoscopically on the visceral pleural surface | One or more radiopaque anchors delineate a point or target region; intraoperative CT verifies the anchor–nodule relationship | Requires superficial visceral-pleural needle passage, radiation exposure, strict anchor accountability, modeling, and hybrid-OR occupation; comparative effectiveness is unestablished | Hybrid OR with intraoperative CT and coordinated multidisciplinary support | Selected deep, poorly palpable, multifocal, or percutaneously inaccessible lesions for which intraoperative spatial verification may be valuable |

**Notes.** The comparison is qualitative. Reported technical success and complication rates should not be compared directly across strategies because studies differ in eligibility criteria, outcome definitions, marker materials, imaging protocols, operator experience, and study design. “Potentially suitable situations” indicate possible applications rather than evidence-based indications. The present workflow is non-percutaneous but is not entirely puncture-free because superficial needle passage through the visceral pleura is required.

**Abbreviations:** CT, computed tomography; ICG, indocyanine green; OR, operating room; 3D, three-dimensional.

**Representative sources for EndNote insertion**

S1. Ichinose J, Kohno T, Fujimori S, et al. Efficacy and complications of computed tomography-guided hook wire localization. Ann Thorac Surg. 2013;96:1203–1208. doi:10.1016/j.athoracsur.2013.05.026.

S2. Hu L, Gao J, Chen C, et al. Comparison between the application of microcoil and hookwire for localizing pulmonary nodules. Eur Radiol. 2019;29:4036–4043. doi:10.1007/s00330-018-5939-4.

S3. Yang F, Zhao H, Sui X, et al. Comparative study on preoperative localization techniques using microcoil and hookwire by propensity score matching. Thorac Cancer. 2020;11:1386–1395. doi:10.1111/1759-7714.13365.

S4. Yang SM, Ko WC, Lin MW, et al. Image-guided thoracoscopic surgery with dye localization in a hybrid operating room. J Thorac Dis. 2016;8(Suppl 9):S681–S689. doi:10.21037/jtd.2016.09.55.

S5. Fang HY, Chen KA, Wen YW, et al. Efficacy and safety of preoperative vs. intraoperative computed tomography-guided lung tumor localization: a randomized controlled trial. Front Surg. 2022;8:809908. doi:10.3389/fsurg.2021.809908.

S6. Luo K, Lin Y, Lin X, et al. Localization of peripheral pulmonary lesions to aid surgical resection: a novel approach for electromagnetic navigation bronchoscopic dye marking. Eur J Cardiothorac Surg. 2017;52:516–521. doi:10.1093/ejcts/ezx114.

S7. Sekimura A, Iwai S, Yamagata A, et al. Virtual thoracoscopic imaging-assisted pleural marking of pulmonary nodules. J Thorac Dis. 2020;12:4148–4156. doi:10.21037/jtd-20-805.

S8. Tang H, Yue P, et al. Three-dimensional printing template for intraoperative localization of pulmonary nodules in the pleural cavity. JTCVS Tech. 2022;16:139–148. doi:10.1016/j.xjtc.2022.10.003.

S9. Yu H, Tian W, Sun Y, et al. Localization of small peripheral pulmonary nodules for surgical resection: a new intraoperative technique in hybrid operating room. J Cardiothorac Surg. 2022;17:241. doi:10.1186/s13019-022-02012-4.
